# Crystalloid resuscitation in trauma patients: deleterious effect of 5L or more in the first 24h

**DOI:** 10.1186/s12893-018-0427-y

**Published:** 2018-11-06

**Authors:** D.G. Jones, J. Nantais, J. B. Rezende-Neto, S. Yazdani, P. Vegas, S. Rizoli

**Affiliations:** 1 Department of Surgery, University of Toronto, 30 Bond Street, Toronto, ON M5B 1W8 Canada; 2 St. Michael’s Hospital Department of Surgery, University of Toronto, 30 Bond Street, Toronto, ON M5B 1W8 Canada

**Keywords:** Fluid resuscitation, Crystalloids, Trauma, Emergency, Outcomes

## Abstract

**Background:**

Over-aggressive intravenous fluid therapy with crystalloids has adverse effects in trauma patients. We assessed the role of large-volume (≥5l) administration of crystalloids within 24h of injury as an independent risk-factor for mortality, in-hospital complications, and prolonged mechanical ventilation.

**Methods:**

A retrospective cohort analysis of adult trauma patients admitted to a level 1-trauma center between December 2011 and December 2012. Patient demographics, clinical and laboratory values, and total resuscitation fluid administered within the first 24h of injury were obtained. Outcomes included mortality, in-hospital complications and ventilator-days. Multivariable logistic regression and Poisson regression analyses were performed to investigate any association between the administration of ≥5L crystalloids with the aforementioned outcomes while controlling for selected clinical variables.

**Results:**

A total of 970 patients were included in the analysis. 264 (27%) received ≥5L of crystalloids in the first 24h of injury. 118 (12%) had in-hospital complications and 337 (35%) required mechanical ventilation. The median age was 46 years (interquartile range (IQR) 27–65) years and 73% (*n* = 708) were males. The median injury severity score (ISS) was 17 (IQR 9–25). Overall mortality rate was 7% (*n* = 67). Multivariable logistic regression analysis showed several variables independently associated with mortality (*p* < 0.05), including resuscitation with ≥5L crystalloid in the first 24h (adjusted odds ratio (aOR) 2.55), older age (aOR 1.03), higher ISS (aOR 1.09), and lower temperature (aOR 0.68). The variables independently associated with in-hospital complications (p < 0.05) were older age, longer ICU stay, and platelet transfusion within 24h of the injury. Need for mechanical ventilation was more common in patients who received ≥5L crystalloids (RR 2.31) had higher ISS (RR 1.02), developed in-hospital complications (RR 1.91) and had lower presenting temperature (RR 0.87).

**Conclusion:**

Large-volume crystalloid resuscitation is associated with increased mortality and longer time ventilated, but not with in-hospital complications such as pneumonia and sepsis. Based on this data, we recommend judicious use of crystalloids in the resuscitation of trauma patients.

## Background

Trauma is a major cause of mortality, with traumatic injures estimated to result in over five million deaths annually worldwide [1]. Severe hemorrhage is responsible for approximately 55% of in-hospital deaths after trauma [2, 3]. Therefore, hypotension and hypoperfusion should be promptly addressed. [4–6] Management of hemorrhagic shock calls for expeditious hemorrhage control, volume resuscitation with crystalloids and blood products, and treatment of coagulopathy. Accordingly, it has been demonstrated that pre-hospital resuscitation with intravenous crystalloid improves survival in trauma patients [6]. Despite the benefits of volume replenishment in trauma previous studies showed that patients with uncontrolled sources of bleeding would likely benefit from low-volume resuscitation [7–10]. However, the ideal volume of crystalloid infusion in hemorrhagic shock is a matter of debate. A recent multicenter randomized controlled trial was unable to show lower mortality with restrictive use of crystalloid fluid and preferential use of blood products in hemorrhagic shock trauma patients [11].

Injudicious use of crystalloid during resuscitation has been shown to exacerbate the systemic insult in response to hemorrhagic shock, including worsened cardiac function, pulmonary complications, gastric dysmotility, abdominal compartment syndrome, coagulopathy, immunological and inflammatory dysfunction [12–14]. Excessive fluid resuscitation is also linked to reperfusion injury and up-regulation of various inflammatory processes indicated by leukocyte adhesion, capillary leakage and edema [3, 15]. In keeping with these principles, a retrospective trial demonstrated a survival advantage in elderly patients receiving limited-volume crystalloid resuscitation [16]. Additionally, a single-centre retrospective trial in the United States demonstrated higher mortality in trauma patients receiving more than 1.5L of crystalloid in the emergency department [17]. In the present study, we set forth to investigate whether large-volume crystalloid resuscitation of the adult trauma patient was associated with higher mortality. We also investigated the incidence of complications and duration of mechanical ventilation.

## Methods

### Study population

A retrospective cohort analysis of all adult trauma patients admitted to St. Michael’s Hospital (SMH) between December 2011 and December 2012 was performed. Patients with traumatic hanging injuries, and those who died within 1h of hospital admission were excluded from data analysis. The study was approved by the research ethics board (REB) of St. Michael’s Hospital, Toronto, Canada.

### Data source

The trauma registry of our institution captures data on individual trauma patients admitted to the hospital’s Emergency Department (ED) up to the day of discharge. The following data were obtained from the registry: demographic information, Injury Severity Score (ISS), initial laboratory parameters (hemoglobin, platelet count, international normalized ratio (INR), partial thromboplastin time (PTT), lactate, pH, fibrinogen), total volume of crystalloid and blood products administered during the first 24h of injury (including pre-hospital phase), intensive care unit (ICU) stay and hospital length of stay (LOS), ventilator days, in-hospital complications, and in-hospital mortality. Data from pre-hospital fluid infusion data were extracted from ambulance report forms. Crystalloid infusion was calculated as the total volume of normal saline (NS) and/or lactated Ringer’s (LR) received during the first 24h, starting from the time of injury. Quality of data was assessed for accuracy and completeness. Any outliers were verified by revisiting patient records. Missing values from the trauma registry were obtained from the hospital charts when available. Due to a high percentage of missing data for pH values (258/903 missing) and lactate values (475/903 missing), these variables were not included in the analysis. Furthermore, blood pressure on arrival was not collected and missing values of base excess (608/903 missing) precluded their inclusion in the analysis.

The primary outcome was in-hospital mortality. This was recorded in the registry as either death during hospital stay or alive at discharge. Secondary outcomes included: development of in-hospital complications (IHC) defined by pneumonia and/or sepsis) and total number of ventilator-days (measured as total number of days the patients required mechanical ventilation). In our study, large-volume resuscitation was defined as infusion of more than 5l of crystalloids in the first 24h.

### Definitions of complications

Complications were defined by the Ontario Trauma Registry Comprehensive Data Set (OTR CDS) data dictionary (May 2014) [18]. The OTR CDS provides detailed information on complications experienced by traumatically injured patients in the province of Ontario (see Appendix for definitions).

### Statistical analysis

Descriptive statistics were performed for all variables. Continuous variables were reported as median and interquartile range (IQR), as the variables did not follow a normal distribution. Categorical variables were reported as absolute numbers and percentages. Univariate in-hospital mortality (non-survivors versus survivors) and complications (complications versus no-complications) were compared using Wilcoxon Rank-Sum test for continuous variables and Chi-square test for categorical variables. To assess the independent relationship of large-volume crystalloid transfusion to mortality and complications, we performed a multivariable logistic regression analysis using a backward selection process while controlling for variables selected a priori. The results were reported as adjusted odds ratio (aOR) with 95% confidence interval (CI).

A multivariable Poisson regression analysis was performed to assess the independent association between administration of ≥5L crystalloid/24h with respect to number of ventilator-days while controlling for variables selected a priori. Prior to multivariable modeling, the variables were assessed for multicollinearity using tolerance statistics. Appropriate numbers of variables were used so as not to overfit our model. Presence of variance inflation factor (VIF) of > 0.4 was considered to be multicollinear. Model diagnostics were performed, and a *p*-value of < 0.05 was considered statistically significant. All data analyses were carried out using Statistical Software SAS (v.9.3 SAS Institute, Carry, NC).

## Results

### Patient characteristics

During the study period, 987 adult trauma patients were admitted to St. Michaels Hospital. A total of 970 patients were included in the final analysis, 17 were excluded because of inadequate data. Patient demographics, fluid resuscitation volume, clinical and laboratory admission parameters and blood product transfusion data are shown in Table 1. Patients were categorized according to the volume of crystalloid: ≥5L or < 5L of crystalloids within 24h of the injury (Table 1). Of the 970 patients, 264 (27%) received ≥5 L of crystalloids in the first 24h following injury while 706 (73%) received < 5L of crystalloids. Apart from gender, all other parameters were significantly different (*p* < 0.05) between the patients according to the volume of crystalloid received. Patients who received ≥5L of crystalloids within 24h were, for the most part, more severely injured (median ISS17 (IQR 9–25)), had worse physiologic parameters during admission, received more blood products (except for cryoprecipitate), had higher complication rate and ultimately higher mortality (Table 1).

**Table 1:** Clinical characteristics of trauma patients included in the analysis (*n* = 970) and patients receiving ≥5L versus < 5L crystalloids in 24 h of injuries

Variable	≥5L crystalloid	< 5L crystalloid	*p*-value
N	264	706	
Gender – male n (%)	194 (73)	514 (73)	0.83
Age – years (range)	43 (26–57)	48 (27–67)	0.02
ISS (range)	22 (16–32)	16 (5–25)	<.0001
Total Crystalloid 24h, L (range)	8.3 (6.1–11.1)	1.5 (0.5–2.7)	<.0001
Total PRBC 24h, U (range)	0.5 (0–4.5)	0 (0–0)	<.0001
Total FFP 24h, U (range)	0 (0–1)	0 (0–0)	<.0001
Total Platelets 24h, U (range)	0 (0–1)	0 (0–0)	<.0001
Temperature, ^0^C (range)	36.2 (35.5–36.7)	36.3 (35.8–36.8)	0.005
Hemoglobin, g/L (range)	131 (117–141)	136 (123–148)	<.0001
Platelet count, 10^9^/L (range)	203 (163–238)	214 (176–256)	0.0024
INR (range)	1.1 (1.1–1.2)	1.1 (1–1.1)	< 0.0002
PTT, seconds (range)	27.2 (25.1–30.7)	28.1 (26–0.3)	0.025
Fibrinogen, g/L (range)	2.1 (1.6–2.5)	2.6 (2.1–3.)	<.0001
PH (range)	7.3 (7.2–7.3)	7.4 (7.3–7.4)	<.0001
Lactate, mmol/L (range)	2.7 (1.8–4.1)	2 (1.5–2.8)	<.0001
LOS, days (range)	10 (4–21)	4 (1–9)	<.0001
ICU days (range)	2 (1–9)	0 (0–1)	<.0001
Ventilator days (range)	2 (0–7)	0 (0–0)	<.0001
Complications n (%)	69 (26)	49 (7)	<.0001
Mortality n (%)	39 (15)	28 (4)	<.0001

Continuous variables presented as medians & interquartile ranges (25th - 75th percentile) (Wilcoxon Rank-Sum test used for analysis). Categorical variables presented as absolute numbers & percentages (Chi-square test used for analysis). Abbreviations: *ICU* intensive care unit, *INR* international normalized ratio, *ISS* injury severity score, *LOS* length of stay, *PRBC* packed red blood cells, *PTT* partial thromboplastin time

### Mortality

Univariate analysis between non-survivors and survivors showed that, aside from gender, all other demographics were significantly different between survivors and non-survivors (Table 2). Physiologic variables were also noted to be worse in the non-survivors group (Table 2).

**Table 2:** Univariate comparison between non-survivors and survivors

Variable	Non-survivors	Survivors	*p*-value
N	67 (7)	903 (93)	
Gender, male n (%)	49 (73)	659 (73)	0.978
Age^a^, years (range)	58 (36–75)	45 (27–63)	0.003
ISS^a^ (range)	27 (25–35)	17 (9–25)	<.0001
Temperature, ^0^C (range)	35.6 (35.8–36.8)	36.3(35.8–36.8)	<.0001
INR ^a^ (range)	1.14 (1.1–1.3)	1.06 (1–1.15)	<.0001
Hemoglobin, g/L (range)	126 (107–141)	135 (123–146)	0.0011
Platelet, 10^9^/L (range)	172 (147–231)	212 (175–254)	0.0001
Fibrinogen, g/L (range)	1.9 (1.2–2.6)	2.4 (2–2.9)	0.0001
PH (range)	7.3 (7.2–7.4)	7.3 (7.3–7.4)	0.0004
Lactate, mmol/L (range)	3.9 (2.3–5.9)	2.2 (1.5–3.1)	<.0001
Total Crystalloid 24h, L ^a^ (range)	5.3 (2.1–10)	2.2 (1–5)	<.0001
LOS, days (range)	2 (1–9)	5 (2–12)	0.0044
ICU days (range)	2 (1–6)	0 (0–2)	<.0001
Ventilator-days (range)	3 (1–6)	0 (0–2)	<.0001
Complications n (%)	16(24)	102(11)	0.002

Continuous variables presented as medians & interquartile ranges (25th - 75th percentile) (Wilcoxon Rank-Sum test used for analysis). Categorical variables presented as absolute numbers & percentages (Chi-square test used for analysis)

^a^Predictors (risk factors) assessed for association with mortality in multivariable regression analysis

The non-survivor group received a median resuscitative crystalloid volume of 5.3L in the first 24h (IQR 2.1–10) versus 2.2 L (IQR 1–5) in the survivor group. Larger volumes of blood products were also administered in the first 24 h to the non-survivor group as compared to the survivor group (Table 2).

Multivariable logistic regression analysis showed that older age, higher ISS, infusion of ≥5L crystalloids within 24 h of injury, and lower temperature were independent predictors of mortality (Table 3). Ultimately, patients receiving ≥5L crystalloids within 24 h of injury died as a result of multi organ dysfunction (MOD) associated with recalcitrant coagulopathy of trauma. The regression model had excellent discrimination (receiver operating characteristic: 0.87) and calibration (Hosmer-Lemeshow goodness-of-fit statistic 0.28).

**Table 3:** Multivariable logistic regression model associated with hospital mortality

Predictor Variable	Adjusted Odds Ratio (95% CI)	Test Statistic	*p*-value
≥5L crystalloid (vs. < 5L)	2.55 (1.38–4.72)	8.86	0.0029
Age	1.03 (1.01–1.04)	13.37	0.0003
ISS	1.09 (1.06–1.12)	36.84	<.0001
Temperature	0.68 (0.54–0.86)	10.03	0.0015

C statistic = 0.87; Hosmer Lemeshow goodness of fit = 0.30

Abbreviations: *ISS* injury severity score

### In-hospital complications

A total of 118 patients (12%) developed complications. Univariate association of selected risk factors with in-hospital complications status are described in Table 4. Patients who experienced in-hospital complications were older (53(IQR 33–65) vs 44(27–64), *p* = 0.004); severely injured (26 (IQR 19–35) vs. 16 [8–17, 19–26], *p* < 0.0001) and received more blood products (PRBCs, platelets and fibrinogen). Total duration of ICU stay (15 (IQR 9–24) vs 0 (IQR 0–1) *p* < 0.0001); duration of mechanical ventilation ((13.5 (8–21)vs0 (0–1), p < 0.0001) and total hospital stay ((26 (IQR 17–45)vs. 4 (IQR 1–9), p < 0.0001) were significantly longer in patients who experienced complications compared to the patients without (Table 4). Furthermore, mortality was significantly higher in patients with complications compared to patients without complications (14% versus 6%, *p* < 0.0001). The variables found to be independently associated with in hospital complications in multivariable logistic regression were: older age (aOR1.03 (95% CI: 1.01–1.05), *p* < 0.001); longer ICU days (aOR 1.58 (1.46–1.7), *p* < 0.0001) and administration of platelet units within 24 h (aOR 1.36 (1.00–1.84), *p* = 0.044) (Table 5).

**Table 4:** Univariate comparison of patients with and without complications

Variable	Complication	No Complication	*p*-value
N	118 (12)	852 (88)	
Gender, male n(%)	89 (75)	619(73)	0.525
Age, years^a^	53 (33–65)	44 (27–64)	0.004
ISS^a^	26 (19–35)	16 (8–25)	< 0.0001
Temperature, ^0^C ^a^	35.9 (35.2–36.6)	36.3 (35.8–36.8)	< 0.0001
INR	1.1 (1.03–1.2)	1.06 (1–1.15)	0.0178
Hemoglobin, g/L	132.5 (117.5–143)	135 (122–147)	0.1388
Platelets, 10^9^/L ^a^	207 (156–238)	211 (173–254)	0.0510
Fibrinogen, g/L	2.1(1.64–2.6)	2.5 (1.99–2.9)	0.0003
pH	7.3(7.3–7.4)	7.6(7.3–7.4)	< 0.0001
Lactate, mmol/L	2.8(1.75–4.45)	2.2 (1.5–3.2)	0.0019
Total Crystalloid 24h^a^, L	6.2 (2.7–10.2)	2.1 (0.9–4.6)	< 0.0001
Total PRBC 24h, U	0 (0–4)	0 (0–0)	< 0.0001
LOS, days^a^	26 (17–45)	4 (1–9)	< 0.0001
ICU days^a^	15 (9–24)	0 (0–1)	< 0.0001
Ventilator-days^a^	13.5 (8–21)	0 (0–1)	< 0.0001
Mortality, n(%)	16 (14)	51 (6)	< 0.0001

Data are presented as medians with interquartile ranges (25th - 75th percentile) for continuous variables and were analysed using Wilcoxon Rank-Sum test. Categorical variables are presented as absolute numbers and percentages and were analysed using Chi-square test. ^a^ Predictors (risk factors) assessed for association with mortality in multivariable regression analysis

**Table 5:** Multivariable logistic regression model associated with complications

Predictor Variable	Adjusted Odds Ratio (95% CI)	Test Statistic	*p*-value
Age	1.03 (1.01–1.05)	10.91	< 0.001
ICU days	1.58 (1.46–1.7)	134.19	< 0.0001
Total platelet (24h)	1.36 (1.00–1.84)	4.05	< 0.044

C statistic = 0.97; Hosmer Lemeshow goodness of fit = 0.27

Abbreviations: *ICU* intensive care unit

### Ventilator-days

A multivariable Poisson regression estimation of the association between patient factors and the need for ventilator support was assessed. Results showed that administration of ≥5L crystalloid in the first 24h was associated with a significant increase in ventilator-days (RR 2.31 (95%CI 1.81–2.96), *p* < 0.0001. Other factors included higher ISS (1.02 (95% CI 1.01, 1.03), p < 0.0001), development of complications (RR1.91 (95%CI 1.48, 2.48), p < 0.0001) and low initial temperature (0.87 (95% CI 0.80, 0.96), *p* = 0.0039) (Table 6).

**Table 6:** Multivariable Poisson regression estimating the association of administration of ≥5L crystalloid/24h of injury and duration of ventilator stay

Risk Factor	RR (95% CI)	Test Statistic	*p*-value
Age	1.00 (0.99, 1.00)	0.28	0.5978
ISS^a^	1.02 (1.01, 1.03)	23.63	< 0.0001
Temperature^a^	0.87 (0.80, 0.96)	8.32	0.0039
Complications^a^	1.91 (1.48, 2.48)	24.53	< 0.0001
Gender, Male	1.14 (0.89, 1.47)	1.13	0.2868
Total PRBC in 24h	0.99 (0.98, 1.00)	1.45	0.2293
≥5 L crystalloid^a^	2.31 (1.81, 2.96)	45.29	< 0.0001

^a^ Patient factors related to need for ventilator support

## Discussion

In our retrospective cohort study, we found that trauma patients who received ≥5L of crystalloids in the first 24 h of injury had significantly higher mortality rates (26%), when compared to patients who received < 5L of crystalloids within the same period (7%). This increase in mortality represents an odds ratio of 2.55. Moreover, ≥5L fluid resuscitation in the first 24 h of injury was an independent predictor of mortality, along with older age, higher ISS, and lower admission temperature, accentuating the overall detrimental effect of injudicious fluid infusion in trauma patients. A previous study showed higher mortality related to increased crystalloid administration in the emergency department [17]. However, that study did not include pre-hospital and total fluid administration in 24 h, which we believe to be an important and potentially less biased measure of total resuscitation.

The importance of fluid resuscitation to maintain tissue perfusion in hemorrhagic shock has been well stablished and should not be underestimated. However, studies showed that resuscitation regimens aimed at decreasing crystalloid infusion and delivering higher ratios of blood products led to fewer complications associated with excessive fluids, less coagulopathy and ultimately increased survival [19–22].

In keeping with those reports, our findings provide further support for a more conservative approach to fluid resuscitation.

At the microvasculature and cellular levels, large volume resuscitation provokes generalized increase in interstitial fluid and cellular edema. These changes have been linked to organ dysfunction [12]. Excessive LR infusion, particularly the D-isomer of lactate, is known to increase the expression of inflammatory genes, neutrophil inflammatory burst, and neutrophil adhesion molecules [23, 24].

In our study, the difference in fluid volume between survivors and non-survivors was more than double. Nonetheless, we speculate that higher volume differences would have had an even more pronounced effect in the parameters assessed in our study [25–27]. Small differences in intravenous fluid volumes between study groups has been a major limitation in studies designed to investigate low-volume resuscitation and permissive hypotension in trauma patients. For instance, a randomized prospective clinical trial terminated prematurely because it failed to demonstrate decreased mortality with intraoperative hypotensive resuscitation at a target mean arterial pressure of 50 mmHg compared to 65 mmHg [28]. The lack of difference in mortality in the aforementioned study could be explained by the modest amounts of intravenous crystalloid infusion overall (median volume of 2–2.2 l) and the negligible difference in the total volumes of fluid received between the groups (approximately 1 l).

As expected, our study showed that patients who developed in-hospital complications had significantly longer ICU and hospital LOS. However, volume infusion within the first 24h was not an independent predictor of the aforementioned findings. Nevertheless, infusion of ≥5L of crystalloids in the first 24h was associated with a significant increase in the duration of mechanical ventilation. Thus, it is conceivable that higher volume infusion could have a primary effect on pulmonary function interfering with weaning from mechanical ventilation. Moreover, other detrimental effects linked to high volume resuscitation also contributed to more prolonged ICU LOS. Accordingly, previous reports showed that mechanical ventilation is a known risk factor for in-hospital complications linked to significant personal and economic costs [8, 29, 30].

The extent of physiologic derangement as reflected in laboratory variables was associated with higher crystalloid resuscitation volume. This was demonstrated by higher levels of INR, PTT, and lactate, together with lower fibrinogen and pH; previous studies corroborate these findings [12, 19]. Younger patients and patients with higher injury severity scores (ISS) also tended to receive more fluids in our study. The effect of intravenous resuscitation volume on mortality of elderly trauma patients compared to nonelderly has been previously addressed [17]. It was shown that mortality was almost three times more likely in elderly patients who received 3 L of crystalloid in the emergency department compared to nonelderly patients receiving similar volumes. In addition, older age (> 80) and Glasgow coma scale (GCS) < 8 were also significantly related to mortality. Certainly, the combination of excessive fluid administration and advanced age can exacerbate complications after resuscitation.

The use of vasopressors in severe trauma patients has been described as an option to decrease fluid resuscitation volumes [31, 32]. However, strong support for the use of this strategy in the exsanguinating patient is still lacking [32]. Additionally, the relationship between cardiac output, volume status, and venous return in the hemorrhaging patient has restricted the use of this strategy [33]. Moreover, concerns have been raised about arteriolar constriction and reduced micro-circulatory flow to the splanchnic circulation with vasopressor therapy in hemorrhagic shock. However, experimental studies suggest that the doses of vasopressor required in hemorrhagic shock resuscitation are, for the most part, insufficient to compromise splanchnic microcirculation [34].

Our study has several limitations inherent in retrospective data collection. Despite a cohort of 970 patients the study population is considerably heterogeneous, even though attempt was made to match these groups as well as possible. However, in a single center study like ours the effect of group differences is considerably important in the data analysis. Another important limitation was the inability to include lactate, pH, admission blood pressure and base excess data in the multivariate logistic regression analysis because of significant missing data. Lactate and base excess are important markers of tissue perfusion, thus a reliable marker of the resuscitation efforts. Furthermore, admission blood pressure of severely injured patients help guide clinical decision making with respect to the need for ongoing volume replacement. Finally, we assessed crystalloid administration during the first 24 h after injury. It is conceivable that the effect of fluids administered during other periods of the hospital admission were not considered in the results. Nonetheless, our objective was to determine the effect of fluids received during the initial 24 h on the outcome of the patients.

## Conclusion

Large-volume fluid resuscitation (≥5L) with crystalloids in the first 24h after traumatic injury is associated with higher mortality rates and prolonged need of mechanical ventilation. In addition, age, higher ISS, and presenting temperature were independently predictive of in-hospital mortality. The current study supports the principles of judicious fluid resuscitation in trauma. These findings also highlight the need for revision of current resuscitation guidelines for fluid administration to traumatically-injured patients.

## Appendix

### OTR CDS definitions for pneumonia and sepsis [29]

**Pneumonia:** Patients with evidence of pneumonia that develops during the hospitalization. Patients with pneumonia must meet at least one of the following two criteria:

Criterion 1. Rales or dullness to percussion on physical examination of chest AND any of the following:

- New onset of purulent sputum or change in character of sputum;
- Organism isolated from blood culture; and/or
- Isolation of pathogen from specimen obtained by transtracheal aspirate, bronchial brushing or biopsy.

Criterion 2. Chest radiographic examination shows new or progressive infiltrate, consolidation, cavitation or pleural effusion AND any of the following:

- New onset of purulent sputum or change in character of sputum;
- Organism isolated from the blood;
- Isolation of pathogen from specimen obtained by transtracheal aspirate, bronchial brushing or biopsy;
- Isolation of virus or detection of viral antigen in respiratory secretions;
- Diagnostic single antibody titer (IgM) or fourfold increase in paired serum samples (IgG) for pathogen; and/or f. Histopathologic evidence of pneumonia.

ICD-10-CA codes: J12.0–J18.9, J95.88.

**Systemic sepsis:** Definitive evidence of infection, plus evidence of a systemic response to infection. This systemic response is manifested by the presence of infection and TWO or more of the following conditions:

1. Temperature higher than 38 °C or lower than 36 °C;
2. Sepsis with hypotension despite adequate fluid resuscitation combined with perfusion abnormalities that may include, but are not limited to, lactic acidosis, oliguria or an acute alteration in mental status. Patients who are on inotropic or vasopressor agents may not be hypotensive at the time that perfusion abnormalities are measured;
3. HR higher than 90 BPM;
4. RR greater than 20 breaths/minute or PaCO2 lower than 32 mmHg (less than 4.3 kPa); and
5. WBC greater than 12,000 cells/mm3, less than 4000 cells/mm3 or greater than 10% immature (band) forms.

ICD-10-CA codes: A40.0–A41.9, A49.9.

## Abbreviations

ED: Emergency Department
ICU: intensive care unit
IHC: in-hospital complications
INR: international normalized ratio
ISS: Injury Severity Score
LOS: length of stay
LR: lactated Ringer’s
NS: normal saline
PTT: partial thromboplastin time
